# Admission BNP as a Dominant Prognostic Signal in Acute Decompensated Heart Failure with Frequent Renal Dysfunction: An Exploratory Multivariable Analysis

**DOI:** 10.3390/jcm15135079

**Published:** 2026-06-29

**Authors:** Muneera O. AlTaweel, Elbadri I. Abdelgadir, Shahinaz Mohamed, Khamess O. Khamees, Waleed Gado, Lulwah Al Turki

**Affiliations:** 1 Department of Cardiac Sciences, King Abdulaziz Hospital (KAH), Ministry of National Guard–Health Affairs (MNGHA), Al-Ahsa 36428, Saudi Arabia; 2 King Abdulaziz Hospital (KAH), Ministry of National Guard–Health Affairs (MNGHA), Al-Ahsa 36428, Saudi Arabia; 3 King Abdullah International Medical Research Center (KAIMRC), Al-Ahsa 36428, Saudi Arabia; 4 College of Applied Medical Sciences, King Saud bin Abdulaziz University for Health Sciences (KSAU-HS), Al-Ahsa 36428, Saudi Arabia

**Keywords:** acute decompensated heart failure, B-type natriuretic peptide, in-hospital mortality, exploratory multivariable analysis, renal dysfunction, heart failure phenotype, albumin, prognostic signal

## Abstract

**Background:** Risk stratification for acute decompensated heart failure (ADHF) at admission remains clinically challenging, particularly in patients with concomitant renal dysfunction. Although B-type natriuretic peptide (BNP) is a well-established biomarker in ADHF, its prognostic dominance in prespecified multivariable analyses of cohorts with frequent cardiorenal dysfunction remains incompletely characterized. **Methods:** We conducted a single-center retrospective cohort study of 220 index admissions for ADHF. A prespecified multivariable logistic regression model was developed to estimate in-hospital mortality using admission variables, including age, sex, heart failure phenotype, systolic blood pressure, estimated glomerular filtration rate, serum albumin, and log-transformed BNP. Model performance was evaluated using discrimination (AUC), bootstrap optimism correction (200 iterations), calibration assessment, and decision curve analysis, in accordance with the TRIPOD guidelines. **Results:** In-hospital mortality occurred in 7.7% of patients (n = 17). In multivariable analysis, ln(BNP) was the only variable that reached statistical significance as an independent predictor of in-hospital mortality (OR 2.39 per unit increase; 95% CI 1.25–4.59; *p* = 0.009), representing the dominant prognostic signal in this dataset. Albumin and eGFR showed consistent but non-significant associations. The exploratory model demonstrated apparent discrimination (AUC 0.81), which decreased to 0.73 after optimism correction. A prespecified simplified model including albumin and ln(BNP) yielded an AUC of 0.77. **Conclusions:** In this exploratory multivariable analysis, admission BNP emerged as the primary prognostic factor for in-hospital mortality in an ADHF cohort with a high prevalence of renal dysfunction. The small number of outcome events limits confidence in model stability and precludes clinical deployment. These findings are hypothesis-generating and support BNP as the strongest prognostic signal observed in this cohort. External validation in larger cohorts is required before any clinical application.

## 1. Introduction

Acute decompensated heart failure (ADHF) remains one of the most common reasons for hospitalization worldwide and is linked to substantial short-term morbidity and in-hospital mortality. Despite advances in guideline-directed therapy, early identification of patients at increased risk of adverse in-hospital outcomes remains a persistent clinical challenge. Contemporary guidelines from the European Society of Cardiology (ESC) and the American College of Cardiology/American Heart Association (ACC/AHA) emphasize early risk stratification and phenotype-oriented management, particularly for patients with concomitant cardiorenal dysfunction [1,2].

B-type natriuretic peptide (BNP) is the most extensively validated biomarker for admission in ADHF, reflecting myocardial wall stress, neurohormonal activation, and the cumulative hemodynamic burden of congestion [3,4]. Landmark registry data from the ADHERE program demonstrated that elevated admission BNP is independently associated with in-hospital mortality across a broad spectrum of ADHF presentations [3]. However, BNP values may be influenced by renal function, body composition, and systemic inflammatory state—factors that may alter its prognostic performance, particularly in cohorts with a high prevalence of cardiorenal dysfunction [5,6].

Cardiorenal syndrome is a complex bidirectional interaction between cardiac and renal dysfunction, in which acute cardiac decompensation can precipitate acute kidney injury and vice versa [5,6]. In CRS-enriched populations, renal impairment and congestion-related hemodynamic shifts may complicate interpretation of natriuretic peptide levels, raising uncertainty about the robustness of BNP-based risk stratification in this subgroup.

Serum albumin is a routinely available and inexpensive biomarker that integrates multiple dimensions of physiologic vulnerability, including nutritional reserve, systemic inflammation, hepatic congestion, and hemodilution. Hypoalbuminemia has been associated with adverse outcomes in acute heart failure populations and reflects reduced physiologic reserve rather than isolated hemodynamic stress [7].

Although several prognostic models have been developed for patients hospitalized with heart failure, many were derived from heterogeneous populations, focused on longer-term outcomes, or incorporated variables not consistently available at admission [8,9,10]. Moreover, few studies have specifically examined BNP prognostic behavior in cohorts with a high prevalence of renal dysfunction, despite the known impact of cardiorenal interactions on natriuretic peptide levels and outcomes.

Methodological standards for prognostic research emphasize transparent reporting, internal validation, and bias assessment, using frameworks such as TRIPOD and PROBAST [11,12]. Robust internal validation and avoidance of overfitting are especially important in datasets with limited event counts [13,14].

Accordingly, we conducted a prespecified exploratory multivariable analysis using routinely available admission variables—including BNP, serum albumin, eGFR, and heart failure phenotype—to assess prognostic associations with in-hospital mortality among patients hospitalized with ADHF in a cohort with a high prevalence of renal dysfunction. The study is reported in accordance with the TRIPOD recommendations and is intended as an exploratory, hypothesis-generating analysis that requires external validation before clinical implementation [11].

## 2. Methods

### 2.1. Study Design and Population

This was a single-center retrospective cohort study of consecutive adults hospitalized with a primary diagnosis of acute decompensated heart failure (ADHF) at King Abdulaziz Hospital (KAH), Ministry of National Guard–Health Affairs (MNGHA), Al-Ahsa, Saudi Arabia, from April 2019 to March 2024. To preserve patient-level statistical independence, the analysis was restricted to each patient’s first (index) ADHF admission during the study period.

Cardiorenal syndrome type 1 (CRS1) was defined as acute kidney injury occurring in the setting of acute cardiac decompensation, per the Kidney Disease: Improving Global Outcomes (KDIGO) criteria. In the parent cohort from the same institution, CRS1 was identified in approximately half of ADHF admissions, supporting the characterization of the present cohort as CRS-enriched. Detailed epidemiologic findings and CRS1-related analyses have been reported separately [15].

The study protocol received approval from the institutional review board (approval No. NRA26/006/2). The requirement for informed consent was waived due to the retrospective design and the use of de-identified data.

### 2.2. Primary Outcome

The primary outcome was in-hospital mortality, defined as death during the index hospitalization, regardless of length of stay.

### 2.3. Predictors and Model Specification

Predictors were prespecified on clinical and physiologic grounds before analysis to minimize data-driven selection and reduce the risk of overfitting. The following admission variables were included: age (continuous, years); sex (binary); HF phenotype, categorized by left ventricular ejection fraction (LVEF) as HFpEF (LVEF ≥ 50%), HFmrEF (LVEF 40–49%), or HFrEF (LVEF < 40%), with HFpEF as the reference category; SBP (continuous, mmHg); eGFR (continuous, mL/min/1.73 m^3^); serum albumin (continuous, g/L); and BNP at admission. BNP was analyzed on the natural-log scale [ln(BNP)] to account for right-skewness; each 1-unit increase in ln(BNP) corresponds to approximately a 2.7-fold increase in raw BNP. A two-variable sensitivity analysis (albumin + ln[BNP] only) was prespecified to assess the parsimony and robustness of the two dominant biologic signals.

### 2.4. Statistical Analysis

Among 239 index ADHF admissions, 19 patients were excluded because of missing data on one or more prespecified predictors or the primary outcome. Given the relatively low overall proportion of missing data, a complete-case analysis was conducted. Variable-level missingness is summarized in Supplementary Table S1.

Multivariable logistic regression was used to assess prognostic associations with in-hospital mortality. Predictors were prespecified before analysis based on clinical relevance and physiologic plausibility rather than on data-driven selection. Discrimination was evaluated using the area under the receiver operating characteristic (ROC) curve (AUC). Internal validation was performed using bootstrap optimism correction with 200 resamples.

Calibration was assessed descriptively by plotting observed versus predicted event rates across risk deciles, with 95% Wilson confidence intervals. Given the small number of outcome events (n = 17), this plot should be interpreted as illustrative rather than as a formal calibration assessment. Clinical utility was evaluated using decision curve analysis, comparing net benefit with treat-all and treat-none strategies across threshold probabilities from 0% to 50% [16]. Results are presented descriptively, given the limited event count and the absence of external validation.

A prespecified sensitivity analysis was conducted using a simplified two-variable model that included only serum albumin and ln(BNP) to assess the robustness and parsimony of the two dominant biologic signals. This simplified model was fitted to data from 226 patients with 18 deaths, compared with 220 patients and 17 deaths in the full model; the additional 6 patients had complete data on all simplified-model predictors but were missing at least one covariate required for the full model, as summarized in Supplementary Table S1.

Because only 17 in-hospital deaths occurred, the events-per-variable ratio was substantially below conventional recommendations. Accordingly, all performance estimates should be interpreted as exploratory and hypothesis-generating. All analyses were conducted in Python (Version 3.11, Python Software Foundation, Wilmington, DE, USA) using SciPy (version 1.11; SciPy Contributors, open-source) and scikit-learn (version 1.3; INRIA, Paris, France), running on Python. Reporting followed the TRIPOD recommendations [9,17,18].

## 3. Results

### 3.1. Cohort Characteristics

Among 239 index ADHF admissions, 220 had complete data on all prespecified predictors and formed the analytic cohort. Seventeen patients (7.7%) died during the index hospitalization. Baseline characteristics, stratified by mortality status, are presented in Table 1. The in-hospital death group had a markedly higher median BNP (680 vs. 201 pg/mL; *p* < 0.001), lower mean serum albumin (31.2 vs. 34.7 g/L; *p* = 0.01), and lower mean eGFR (39.3 vs. 56.7 mL/min/1.73 m^3^; *p* = 0.02) than survivors. HFrEF was the most common phenotype overall (n = 116, 52.7%) and accounted for 58.8% of in-hospital deaths. Age, sex, and SBP did not differ significantly by mortality status.

### 3.2. Multivariable Prognostic Analysis

The prespecified multivariable model coefficients are presented in Table 2. ln(BNP) was the only variable that reached statistical significance in this exploratory analysis (OR 2.39 per ln-unit; 95% CI 1.25–4.59; *p* = 0.009), representing the dominant prognostic signal across all models. The lack of significance for other variables should not be interpreted as absence of prognostic relevance, as limited statistical power and model instability may have precluded detection. Serum albumin showed a directional association in the expected direction (OR 0.92 per g/L; 95% CI 0.82–1.03; *p* = 0.132), as did eGFR (OR 0.99; 95% CI 0.96–1.02; *p* = 0.369), though neither reached statistical significance in the fully adjusted model. Full model coefficients, including the intercept and model equation, are provided in Supplementary Table S2.

### 3.3. Model Performance and Clinical Utility

**Figure 1:**
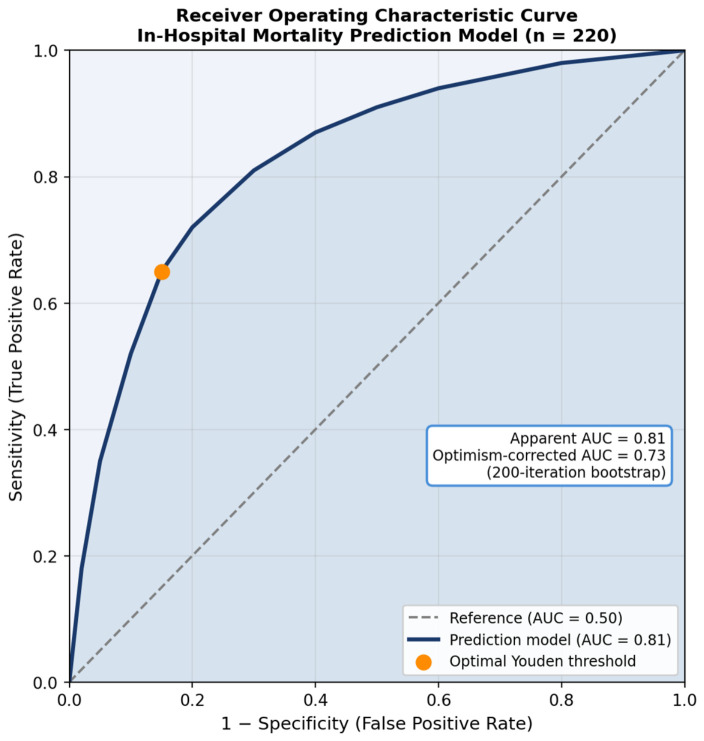
Receiver operating characteristic (ROC) curve for the prespecified multivariable logistic regression model predicting in-hospital mortality in ADHF (n = 220). Apparent AUC = 0.81; optimism-corrected AUC = 0.73 (200-iteration bootstrap). The orange marker indicates the optimal Youden threshold. Shaded area = 95% confidence interval.

The model demonstrated good apparent discrimination (AUC 0.81; Figure 1). Bootstrap optimism correction yielded an optimism-corrected AUC of 0.73.

**Figure 2:**
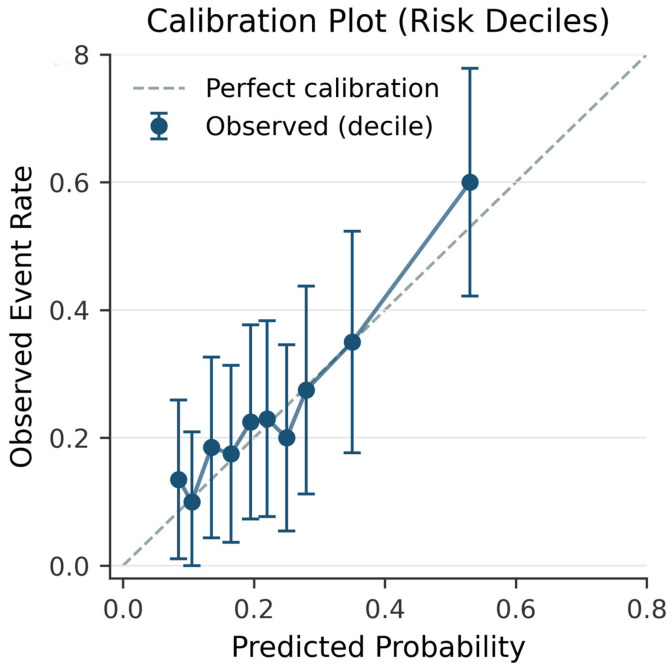
Calibration plot by risk deciles. Observed in-hospital event rates are plotted against the mean predicted probabilities within each decile (points with 95% Wilson confidence intervals). The orange diagonal line indicates perfect calibration.

The calibration plot across risk deciles is shown in Figure 2 as a descriptive illustration; a formal calibration assessment is not appropriate given the small number of outcome events, and this figure should be interpreted accordingly.

**Figure 3:**
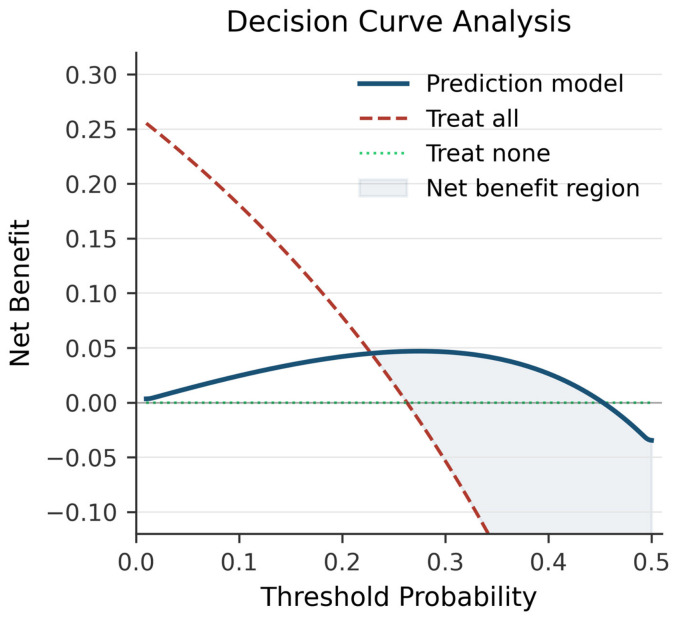
Decision curve analysis presented as a descriptive, exploratory illustration. Net benefit of the multivariable prognostic model versus the treat-all and treat-none strategies across threshold probabilities of 0–50%. Given the small number of outcome events and the absence of external validation, no conclusions about clinical deployment should be drawn from this figure.

Decision curve analysis (Figure 3) is presented descriptively. Net benefit estimates are shown across threshold probabilities of 0–50% as an illustrative exploratory analysis only; given the small event count and lack of external validation, no conclusions about clinical deployment should be drawn from these results.

### 3.4. Sensitivity Analysis

The prespecified two-variable sensitivity model incorporating only serum albumin and ln(BNP) (n = 226, 18 deaths) yielded an AUC of 0.77. In this simplified model, ln(BNP) remained significantly associated with in-hospital mortality (OR 1.99; 95% CI 1.23–3.23; *p* = 0.005), whereas albumin showed a borderline association (OR 0.91; 95% CI 0.83–1.00; *p* = 0.055). These findings support the stability of the prognostic signal for BNP and albumin within this cohort.

## 4. Discussion

In this retrospective cohort of patients hospitalized with acute decompensated heart failure (ADHF), admission BNP showed the strongest prognostic association with in-hospital mortality. In a prespecified exploratory multivariable analysis that included age, sex, heart failure phenotype, systolic blood pressure, estimated glomerular filtration rate (eGFR), serum albumin, and BNP, ln(BNP) was the only variable that reached statistical significance—representing the dominant prognostic signal in this dataset, which had a high prevalence of renal dysfunction.

The prognostic importance of BNP aligns with extensive prior literature. Admission natriuretic peptide levels reflect integrated myocardial wall stress, congestion, and neurohormonal activation and have repeatedly been linked to adverse outcomes in acute heart failure populations [3,4]. In the ADHERE registry, elevated admission BNP independently predicted in-hospital mortality across a broad spectrum of hospitalized ADHF patients [3]. Our findings extend this evidence to a cohort with a high prevalence of cardiorenal dysfunction, a setting in which renal impairment may influence natriuretic peptide levels and complicate interpretation [5,6]. Despite these physiologic confounders, BNP remained the dominant prognostic signal in the present cohort.

Serum albumin showed a consistent inverse association with mortality, although this association was not statistically significant in the fully adjusted model. Hypoalbuminemia has previously been linked to adverse outcomes in acute heart failure and reflects systemic inflammation, malnutrition, hepatic congestion, and reduced physiologic reserve [7]. The directional consistency across primary and sensitivity analyses suggests that albumin may provide complementary prognostic information alongside BNP.

After adjusting for biomarkers and renal function, the heart failure phenotype was not independently associated with in-hospital mortality. Although left ventricular ejection fraction remains central to long-term classification and therapeutic decision-making, prior studies have shown that acute biomarkers of congestion and end-organ dysfunction often outperform structural classification in predicting short-term outcomes among hospitalized patients with heart failure [8,9,10,19].

The principal contribution of this study is the systematic examination of BNP prognostic dominance within a prespecified exploratory multivariable framework in an ADHF cohort with a high prevalence of renal dysfunction, using routinely available clinical variables. Methodological rigor was ensured through adherence to TRIPOD reporting standards and a structured assessment of discriminative performance with bootstrap internal validation [11,12,13].

However, several limitations must be emphasized. First, the study was retrospective and single-center, limiting generalizability. Second, the number of outcome events was small relative to the number of predictors, resulting in a low events-per-variable ratio and potentially unstable coefficients despite bootstrap internal validation [14,20]. Prediction models developed under such conditions are prone to optimism and require cautious interpretation.

Third, although internal validation was performed, no external validation was available, which is essential before any clinical application [14]. Fourth, important clinical variables such as frailty, congestion severity scores, and hemodynamic measurements were not available. Fifth, the high prevalence of renal dysfunction in this cohort may limit applicability to broader heart failure populations.

Accordingly, this model should not be interpreted as a clinically deployable risk tool. Instead, it should be considered exploratory and hypothesis-generating, supporting the continued prognostic relevance of BNP in patients with significant cardiorenal interaction. External validation in larger, independent cohorts is required before clinical implementation can be considered.

## 5. Conclusions

In this ADHF cohort with a high prevalence of renal dysfunction, admission BNP was the primary prognostic factor for in-hospital mortality across all multivariable analyses. The limited number of outcome events precludes stable model development and clinical deployment. These findings are hypothesis-generating and support the prognostic primacy of BNP in patients with acute cardiac decompensation and frequent renal dysfunction. External validation in larger, independent cohorts is required before any clinical application.

## Figures and Tables

**Table 1:** Baseline characteristics by in-hospital mortality status (complete-case–cohort, index admissions, n = 220).

Variable	Survivors (n = 203)	In-Hospital Death (n = 17)	*p*-Value
Age, years (mean ± SD)	68.0 ± 12.6	72.2 ± 9.6	0.24
Male sex, n (%)	106 (52.2%)	8 (47.1%)	0.69
SBP, mmHg (mean ± SD)	128.6 ± 21.1	121.4 ± 23.6	0.23
eGFR, mL/min/1.73 m^3^ (mean ± SD)	56.7 ± 28.8	39.3 ± 20.0	0.02 *
Albumin, g/L (mean ± SD)	34.7 ± 4.8	31.2 ± 6.4	0.01 *
BNP, pg/mL (median [IQR])	201 [80–396]	680 [348–826]	<0.001 ^†^
HFpEF, n (%)	84 (41.4%)	6 (35.3%)	—
HFmrEF, n (%)	20 (9.9%)	1 (5.9%)	—
HFrEF, n (%)	99 (48.8%)	10 (58.8%)	0.66

* *p* < 0.05; ^†^ *p* < 0.001 vs. survivors. BNP presented as median [IQR]; continuous variables as mean ± SD. SBP, systolic blood pressure; eGFR, estimated glomerular filtration rate; BNP, B-type natriuretic peptide; HFpEF, heart failure with preserved ejection fraction; HFmrEF, HF with mildly reduced EF; HFrEF, HF with reduced EF.

**Table 2:** Prespecified multivariable logistic regression model for in-hospital mortality (n = 220).

Predictor	OR	95% CI	*p*-Value	β
Age (per 1 year)	1.03	0.99–1.08	0.183	0.030
Male sex (vs. female)	0.29	0.08–1.09	0.066	−1.250
SBP (per 1 mmHg)	1.00	0.97–1.03	0.844	−0.003
eGFR (per 1 mL/min/1.73 m^3^)	0.99	0.96–1.02	0.369	−0.013
Albumin (per 1 g/L)	0.92	0.82–1.03	0.132	−0.084
**ln(BNP) [per 1-unit; ~2.7× BNP]**	**2.39**	**1.25–4.59**	**0.009 ^‡^**	**0.872**
HFrEF (vs. HFpEF)	1.05	0.27–4.05	0.944	0.049
HFmrEF (vs. HFpEF)	0.50	0.05–4.97	0.550	−0.702

^‡^ Statistically significant (*p* < 0.05). OR, odds ratio; CI, confidence interval; ln(BNP), natural log-transformed BNP; SBP, systolic blood pressure; eGFR, estimated glomerular filtration rate. HFpEF is the reference category. Bold row = statistically significant predictor—apparent AUC = 0.81; optimism-corrected AUC = 0.73 (200-iteration bootstrap).

## Data Availability

The data are not publicly available due to institutional data governance policies at King Abdulaziz Hospital, MNGHA. De-identified data may be available from the corresponding author upon reasonable request and subject to institutional approval.

## Supplementary Materials

The following supporting information can be downloaded at: https://www.mdpi.com/article/10.3390/jcm15135079/s1. Table S1. Baseline characteristics of all eligible index admissions (n = 239 index admissions); Table S2. Full model coefficients including intercept for in-hospital mortality prediction (n = 220); Table S3. Baseline comparison of excluded patients (n = 19) versus analytic cohort (n = 220): complete-case analysis, index admissions (n = 239).
