# Supplementary material for: Admission BNP as a Dominant Prognostic Signal in Acute Decompensated Heart Failure with Frequent Renal Dysfunction: An Exploratory Multivariable Analysis
Source: J Clin Med. 2026 Jun 29;15(13):5079. doi: 10.3390/jcm15135079 (PMC13362842; doi:10.3390/jcm15135079)
Supplement: Supplementary file 1 [file jcm-15-05079-s001.zip › jcm-4346055-supplementary.pdf]

Supplementary Table S1. Missingness among prespecified predictors and outcome (overall cohort, n = 239 index admissions).

Variable	Missing, *n* (%)
Age	0 (0%)
Sex	0 (0%)
SBP	0 (0%)
HF phenotype	0 (0%)
eGFR	1 (0.4%)
Albumin	1 (0.4%)
BNP	9 (3.8%)
In-hospital death (outcome)	4 (1.7%)

Supplementary Table S2. Full model coefficients including intercept for in-hospital mortality prediction (n = 220).

Predictor	β	OR	95% CI Low	95% CI High	*p*-value	
Intercept	-5.275	0.005	0.000	6.088	0.144	
Male (vs. female)	-1.250	0.287	0.076	1.087	0.066	
HFrEF (vs. HFpEF)	0.049	1.050	0.272	4.049	0.944	
HFmrEF (vs. HFpEF)	-0.702	0.495	0.049	4.969	0.550	
Age	0.030	1.031	0.986	1.077	0.183	
SBP	-0.003	0.997	0.969	1.026	0.844	
eGFR	-0.013	0.988	0.961	1.015	0.369	
Albumin	-0.084	0.919	0.824	1.026	0.132	
**ln(BNP)**	**0.872**	**2.392**	**1.246**	**4.593**	**0.009**	

Sensitivity analysis: A prespecified two-variable model (albumin + ln[BNP] only; n = 226, 18 deaths) yielded AUC 0.77, with ln(BNP) OR 1.99 (95% CI 1.23–3.23; p = 0.005) and albumin OR 0.91 (95% CI 0.83–1.00; p = 0.055), confirming robustness of both markers.

Supplementary Table S3. Baseline comparison of excluded patients (n = 19) versus analytic cohort (n = 220): complete-case analysis, index admissions (n = 239).

Variable	Excluded (n = 19)	Included (n = 220)	*p*-value
**Continuous variables (mean ± SD)**			
Age, years	64.9 ± 10.5	68.3 ± 12.4	0.311
SBP, mmHg	135.9 ± 20.4	128.0 ± 21.3	0.120
eGFR, mL/min/1.73 m²	44.0 ± 23.6	55.3 ± 28.6	0.103
Albumin, g/L	33.9 ± 6.1	34.4 ± 5.0	0.670

**Categorical variables, n (%)**			
Male sex	10 (52.6%)	114 (51.8%)	0.946
HFrEF	7 (36.8%)	109 (49.5%)	0.407
HFpEF	8 (42.1%)	90 (40.9%)	
HFmrEF	4 (21.1%)	21 (9.5%)	
Hypertension	18 (94.7%)	202 (91.8%)	0.821
Diabetes mellitus	15 (78.9%)	182 (82.7%)	0.613
CRS1 (AKI during admission)	15 (78.9%)	89 (40.5%)	**0.042***
**Missing variable pattern, n (%)**			
BNP missing	9 (47.4%)	0 (0%)	—
Age missing	5 (26.3%)	0 (0%)	—
Outcome (in-hospital death) missing	4 (21.1%)	0 (0%)	—
eGFR missing	1 (5.3%)	0 (0%)	—
Albumin missing	1 (5.3%)	0 (0%)	—

* $p < 0.05$. CRS1 was the only characteristic that differed significantly between excluded and included patients (78.9 % vs. 40.5 %; $p = 0.042$), likely reflecting higher BNP missingness in more acutely ill CRS1 patients — a form of informative missingness that should be acknowledged as a limitation. All other baseline characteristics (age, sex, SBP, eGFR, albumin, HF phenotype, hypertension, diabetes) did not differ significantly between groups, supporting the overall validity of the complete-case approach. SBP = systolic blood pressure; eGFR = estimated glomerular filtration rate; HFrEF/HFpEF/HFmrEF = heart failure with reduced/preserved/mildly reduced ejection fraction; CRS1 = cardiorenal syndrome type 1.
